# Surgical outcomes of infectious spondylitis after vertebroplasty, and comparisons between pyogenic and tuberculosis

**DOI:** 10.1186/s12879-018-3486-x

**Published:** 2018-11-12

**Authors:** Jen-Chung Liao, Po-Liang Lai, Lih-Hui Chen, Chi-Chien Niu

**Affiliations:** Department of Orthopedics Surgery, Bone and Joint Research Center, Chang Gung Memorial Hospital, Chang Gung University, No.5, Fu-Shin Street Kweishian, Taoyuan, 333 Taiwan

**Keywords:** Vertebroplasty, Infection, Pyogenic, Tuberculosis, Surgery

## Abstract

**Background:**

Infection after vertebroplasty (VP) is a rare but serious complication. Previous literatures showed most pathogens for infection after VP were bacteria; tuberculosis (TB) induced infection after VP was extremely rare. We reported our treatment experiences of cases with infectious spondylitis after VP, and compared the differences between developed pyogenic and TB spondylitis.

**Methods:**

From January 2001 to December 2015, 5749 patients ***had*** undergone VP at our department were reviewed retrospectively. The causative organisms were obtained from tissue culture of revision surgery. Parameters including type of surgery, the interval between VP and revision surgery, neurologic status, and visual analog scale (VAS) of back pain were recorded. Laboratory data at the time of VP and revision surgery were collected. Charlson comorbidity index (CCI), preoperative bacteremia, urinary tract infection (UTI), pulmonary TB history were also analyzed.

**Results:**

Eighteen patients were confirmed with developed infectious spondylitis after VP (0.32%, 18/5749). Two were male and 16 were female. The median age at VP was 73.4 years. Nine patients were TB and the other nine patients were pyogenic. The interval between VP and revision surgery ranged from 7 to 1140 days (mean 123.2 days). The most common type of revision surgery was anterior combined with posterior surgery. Seven patients developed neurologic deficit before revision surgery. Three patients died within 6 months after revision surgery, with a mortality of 16.7%. Finally, VAS of back pain was improved from 7.4 to 3.1. Seven patients could walk normally, the other 8 patients had some degree of disability. Both pyogenic and TB group had similar age, sex, and CCI distribution. The interval between VP and revision surgery was shorter in the patients with pyogenic organisms (75.9 vs 170.6 days). At revision surgery, WBC and CRP were prominently elevated in the pyogenic group. Five in the pyogenic group had UTI and bacteremia; five in TB group had a history of lung TB.

**Conclusions:**

Infection spondylitis after VP required major surgery for salvage with a relevant part of residual disability. Before VP, any bacteremia/UTI or history of pulmonary TB should be reviewed rigorously; any elevation of infection parameters should be scrutinized strictly.

## Background

Since Galibert et al. first demonstrated percutaneous vertebroplasty (VP) for the treatment of haemangioma of the spine in 1987 [1], VP has been generally accepted for the surgical treatment of compression fractures in thoracic or lumbar vertebrae. Reported acute symptomatic complications associated with VP are low: usually 1% for osteoporotic fractures, and up to 10% for metastatic lesion [2, 3]. Although the incidence is very low, infection might indeed occur after VP. Infectious spondylitis after VP is serious, and might lead to neurological deficit and even death. Most infectious pathogens found after vertebroplasty were bacterial; but the largest series studying spinal infection after vertebroplasty or kyphoplasty contained only nine patients [4]. Tuberculosis (TB) after VP is rare; these cases are reported sporadically in the literature [5–10]. There is no literature discussing the differences between pyogenic and TB infection after VP. In this study, we report our experiences in managing patients with infectious spondylitis after VP. The pathogens of these patients included various bacteria or TB. We use their clinical history, laboratory data, and surgical outcomes to uncover differences between pyogenic and TB infectious spondylitis after VP.

## Methods

After obtaining approval from the Institute of Review Board, we retrospectively reviewed patients who had undergone VP in our department for osteoporotic compression fractures between January 2001 and December 2015. The only criteria for enrolment was that causative organisms for infectious spondylitis after VP must be obtained from tissue culture in revision surgery. Patients were categorized into two groups according to the pathogens of their infection after VP: a pyogenic and a TB group. Demographic data was collected from medical records, including age, sex, the number and level of VP, interval between VP and revision surgery, and type of revision surgery. Laboratory data focused on inflammatory parameters including the WBC, ESR, and CRP recorded at the time of VP and revision surgery. The medical condition of these patients was evaluated using the weighted Charlson Comorbidity Index (CCI) [11]. Neurologic status just before revision surgery was determined using the American Spinal Injury Association (ASIA) impairment scale. Back pain status was recorded using the Visual Analogue Scale (VAS) at revision surgery, and final follow up was recorded. We determined final clinical outcomes through ambulation, with four grades: normal ambulation without support, ambulation with crutches or walker support, ambulation in a wheelchair, or unable to ambulate. We especially focused on the incidence of risk factors that might induce the development of infectious spondylitis after VP. These risk factors included urinary tract infection (UTI), any episode of bacteraemia within the 3 months before VP, any episode of organ infection by bacteria or TB within the 3 months before VP, or a history of pulmonary TB.

## Results

Five thousand seven hundred forty nine patients underwent VP in our department in the period of our study. Totally twenty-three patients underwent revision surgeries because they were suspected to have infectious spondylitis after VP by clinical symptoms, laboratory data and radiographic finding; the indications for these 23 patients were intractable back pain with or without neurologic deficit. Eighteen patients were enrolled in to study because they were confirmed to have developed infectious spondylitis at the level of VP according to positive culture data from the revision surgery, the other 5 patients were excluded. The positive culture rate was 78% (18/23) in revision surgeries. The incidence of infectious spondylitis after VP was 0.32% (18/5749) by positive tissue culture. There were 24 vertebrae (10 in thoracic spine and 14 in lumbar spine) involved by infection in these 18 patients. Nine patients had bacterial infection and the other nine patients were confirmed with TB infection. The average age of the 18 patients at the time of VP was 73.4 years (63 to 90) and the mean CCI was 1.7 (0 to 4); the mean interval between VP and revision surgery was 123 days (7 to 1140). The average WBC was within normal range at the time of VP and revision surgery, but CRP and ESR were elevated in most patients, especially at the time of revision surgery. Six patients developed neurologic deficits before revision surgery (one with ASIA B, two with ASIA C, and three with ASIA C). Three patients had treatment with only posterior decompression and instrumentation surgeries, four patients underwent anterior debridement and fusion surgeries, and the other 11 patients underwent combined anterior and posterior surgeries. Just before revision surgery, VAS of back pain was high, at 7.0 points (5 to 9), and eventually improved to 2.7 points (1 to 6). By the final follow up, three patients had died due to surgical and infectious complications; two patients needed wheelchairs for ambulation, four patients could ambulate with the support of walkers, and the other nine patients ambulated normally without any support. The demographic, laboratory, and clinical data of these 18 patients is listed in Table 1.

**Table 1:** Demographic, Laboratory, History, and Clinical Results

Case No.	Age	Causative Organism	Level	1st surgery			Revision surgery			Interval 1st& revision surgery	Revision method	Neurologic Status (ASIA)	Pre-revision Back pain VAS	Final VAS	Final activity	UTI	Bacteremia	Pulmonary TB	CCI
				WBC (X1000(/mL)	ESR (mm/h)	CRP (mg/L)	WBC (X1000)(/mL)	ESR (mm/h)	CRP (mg/L)										
1	80s	Salmonella. enterica	L2, L3	7.6	–	42	7.3	33	4.2	142	P	E	8	2	normal	n	y	n	0
2	60s	*E. coli*	L2, L3, L4	10.1	–	152	18.2	31	99	7	P	E	9	5	normal	y	y	n	4
3	60s	Coag(−) Stapylococcus	L2	15.3	137	139	12.3	119	118.9	35	A + P	E	7	6	normal	y	y	n	3
4	70s	E. Coli	T12	8	–	–	12	29	50	74	A + P	C	7	4	wheelchair	y	n	n	1
5	70s	Peptpstreptococcus	L3	7.4	–	11.3	11.8	70	238	12	A	E	8	–	dead	y	n	n	3
6	70s	Propionibacterium	T11	7.5	–	–	9.2	53	38	175	A + P	D	6	2	walker	n	n	n	1
7	60s	Enterococcus faecalis	T12	10.9	–	62.5	12	99	165	69	A	E	5	5	normal	y	y	n	2
8	70s	Salmonela choleraesuiss	L2	6.3	20	1.3	5.1	62	69.4	122	A	E	7	2	normal	n	y	n	1
9	70s	*Staphylococcus aureus*	L2	11.4	–	–	11.3	54	7.9	47	A + P	E	8	–	dead	n	n	n	2
10	70s	mycobacterium	T9	7.2	24	3	5.7	68	11.4	7	A	B	7	–	dead	n	n	y	1
11	79 s	mycobacterium	T11,T12	7.2	26	49	8.2	26	50.2	15	A + P	E	7	4	walker	n	n	y	3
12	60s	mycobacterium	L4	6.2	–	–	4.9	35	4.9	63	A + P	E	8	2	normal	n	n	n	2
13	70s	mycobacterium	L1	3	21	11	4.2	33	33.2	129	A + P	E	8	2	normal	n	n	n	2
14	70s	mycobacterium	T9,T10	5.3	34	–	11.5	50	119.6	87	P	C	8	3	wheelchair	n	n	y	1
15	70s	mycobacterium	L1	7	–	–	6.4	56	42.4	6	A + P	D	9	2	walker	n	n	y	0
16	70s	mycobacterium	L1	8	50	71	5.5	31	31.5	34	A + P	D	8	1	walker	n	n	n	2
17	70s	mycobacterium	L4	3.8	–	–	8.2	39	3.2	1140	A + P	E	6	4	walker	n	n	n	1
18	90s	mycobacterium	T8,T9	7	65	4.3	9.7	95	115	54	P	C	8	3	wheelchair	n	n	y	1

Abbreviations: *TB* tuberculosis, *N* number, *M* male, *F* female, *T* thoracic, *L* lumbar, *WBC* white blood-cell count, *ESR* erythrocyte sedimentation rate, *CRP* C-reaction protein, Revision method A = anterior debridement +/− reconstruction surgery; *P* posterior instrumentation +/− decompression surgery, *ASIA* American Spinal Cord Injury Association (grade A, B, C, D, E), *VAS* visual analog scale, *CCI* the Charlson Comorbidity index

### The TB group (nine cases)

**Fig. 1:**
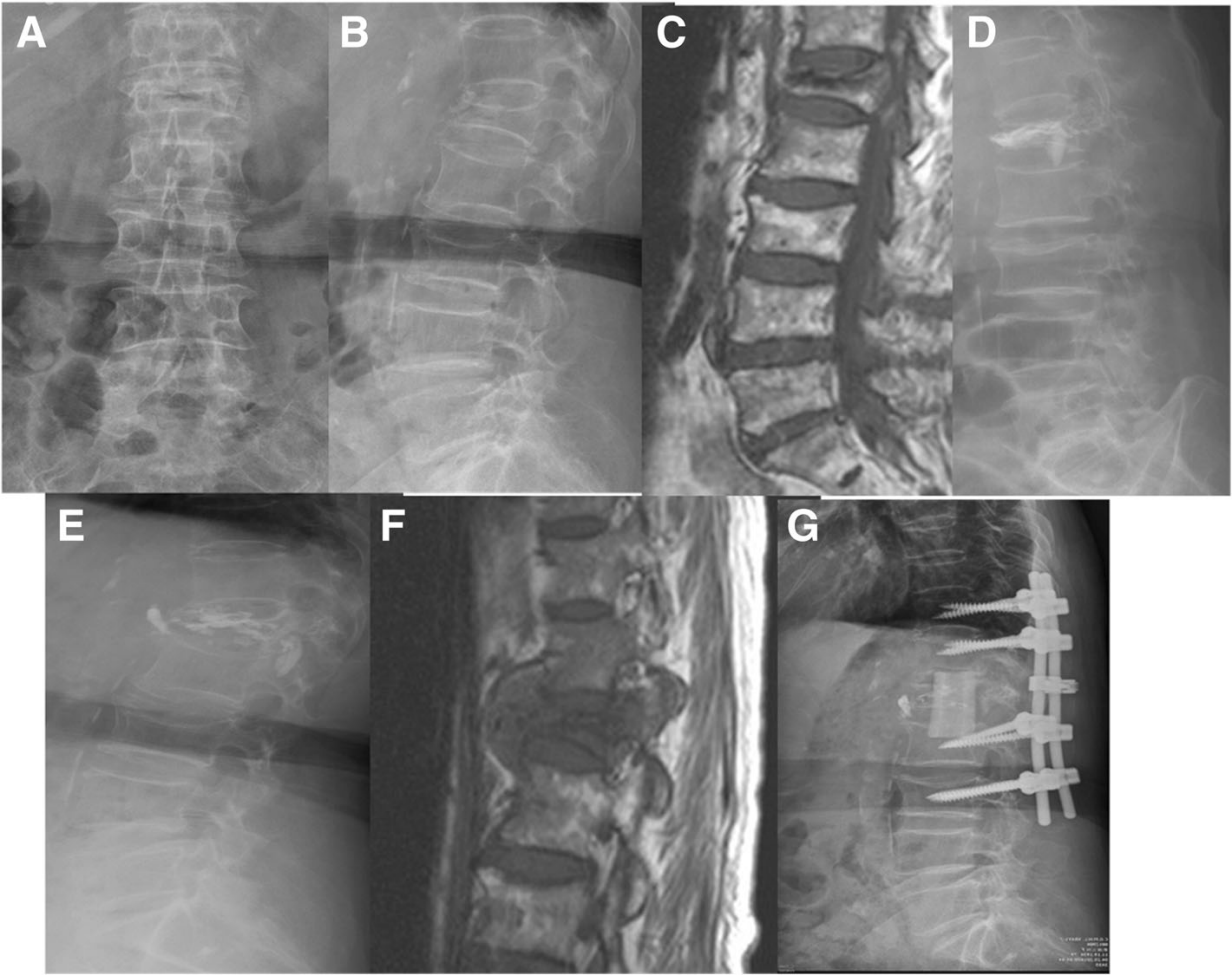
A case represented tuberculosis infection after vertebroplasty (VP). Initial radiographic images and MRI revealed L1 compression fracture (**a**, **b**, **c**). This patient received L1 VP first (**d**). After VP, back pain was subsided dramatically, however, severe low back pain without neurologic deficits was appeared again four months later. Plain radiograph showed L1 body collapse with cement breakage (**e**). MRI revealed L1 spondylitis with abscess formation (**f**). Reconstruction revision surgery was performed for this patient (**g**). Tissue culture showed mycobacterium tuberculosis

Eight female and one male patients were enrolled in this group, with a mean age of 75.1 years (66 to 90). Co-morbidities were found in eight of the nine patients, with an average of 1.2 point of CCI. Five patients had a history of pulmonary tuberculosis. Before VP, WBC counts were all within the normal range; CRP was elevated in only two patients, and was within normal ranges or not checked for the other seven. Before revision surgery, the WBC counts of most patients were slightly elevated, but still within normal range, however the CRP data of seven patients was elevated above the normal limit, with an average of 57.6. The interval between VP and revision surgery ranged from 5 to 1125 days, with a mean of 250 days. At revision surgery, seven patients underwent anterior debridement and reconstruction surgery, six of whom also received posterior instrumentation for augmentation; two patients received posterior only surgeries with decompression, fusion and instrumentation. At the end of follow-up, one patient with paraplegia had passed away, two were in wheelchairs, four patients required a walker, and two had improved functionally and could walk unassisted. Figure 1 showed a case with TB spondylitis after VP. Briefly, a patient with L1 compression fracture after a falling accident underwent VP; L1 spondylitis was diagnosed 4 months later due to back pain again. Combined anterior and posterior surgery was performed, and TB-PCR and culture obtained during the operation were positive.

### The pyogenic group (nine cases)

**Fig. 2:**
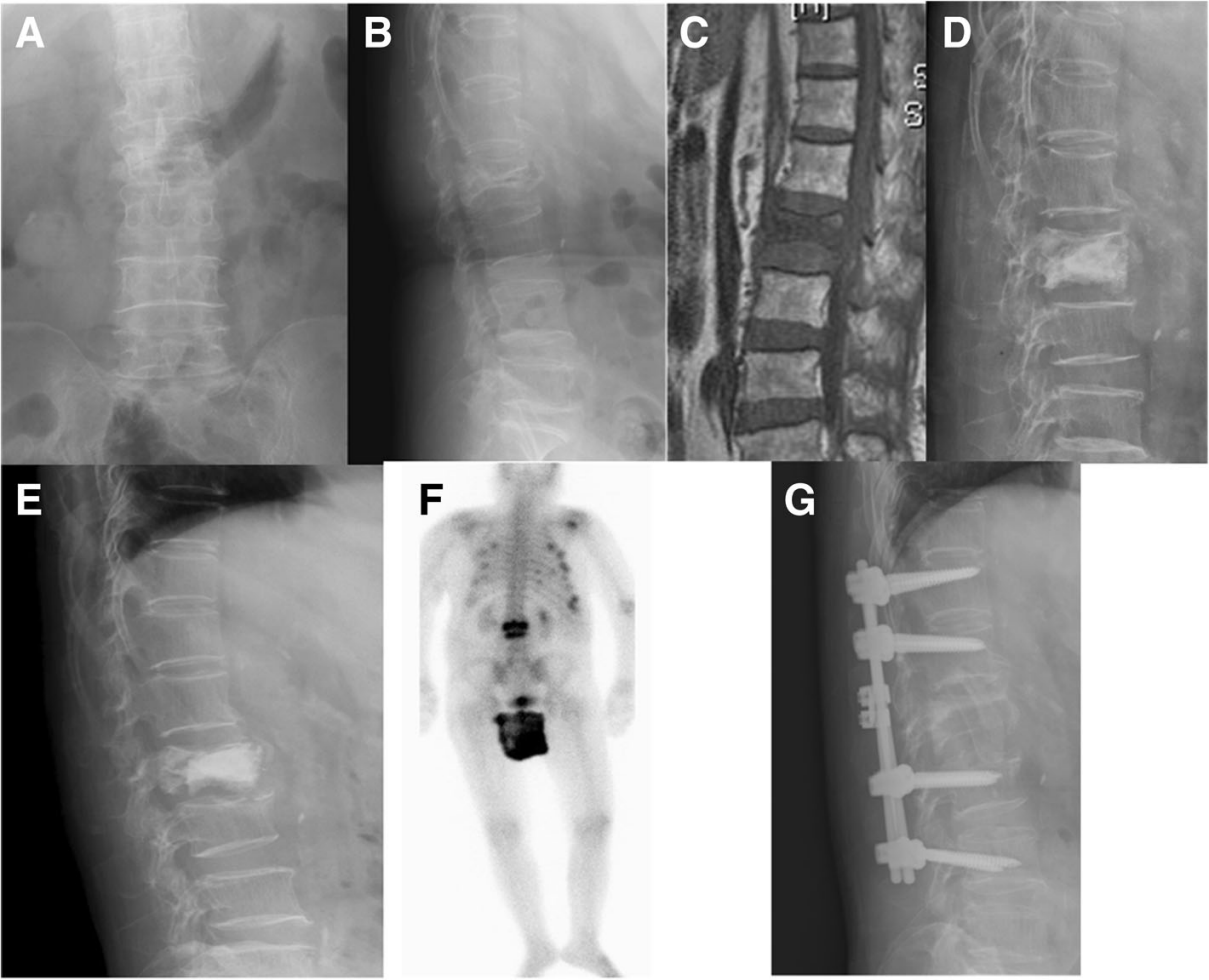
A case represented pyogenic spondylitis after VP. Initial radiographic images and MRI revealed L2 compression fracture (**a**, **b**, **c**). L2 VP was performed first (**d**). Svere low back pain was appeared again one month later. Plain radiograph showed L2 collapse with L2 lower endplate erosion (**e**). Gallium scan revealed L2–3 infectious spondylitis (**f**). Reconstruction revision surgery was performed (**g**). Tissue culture showed Coag (−) Staphylococcus

There were also nine cases in the pyogenic group, including one male and eight females, with a mean age of 71.8 years at the time of VP. Eight patients in this group had underlying disease, with an average CCI of 1.9 in these nine patients. WBC counts were still within normal range (9400/ml) just before VP, but reached the upper limit; six cases had CRP, with an average of 68.0 mg/L. The interval between VP and revision surgery ranged from 7 to 175 days, with a mean of 75.9 days. At the time of revision surgery, the neurologic status of seven patients was intact, two had some degree of neurologic deficit. The mean VAS of back pain was 7.2 points. Three patients received revision surgery by anterior approach only with debridement and fusion; two cases had posterior only surgery with decompression and instrumentation; the other four underwent salvage surgeries using combined anterior and posterior procedures. The causative organisms were various, included *E. coli*, Salmonella enterica, Salmonella choleraesuis, *Staphylococcus aureus*, Coag(−) Staphylococcus, Peptostreptococcus, Propionibacterium, and Enterococcus faecalis. Two patients died, one needed a wheelchair and one patient required a walker for ambulation. The other five improved functionally and could walk unassisted. The average VAS in the survival patients improved to 2.9. Figure 2 demonstrated a case with pyogenic infectious spondylitis after VP.

### Pyogenic versus TB

Both groups had similar age, sex, and CCI distribution. Preoperative neurologic status and VAS scale before revision surgery was also equal. The interval between VP and revision surgery was shorter in the patients with pyogenic organisms (75.9 vs 170.6 days). At revision surgery, WBC and CRP were higher elevated in the pyogenic group. Two in pyogenic group and one in TB group died because of complications by revision surgeries. Final surgical outcomes including VAS and activity in those survivors were similar in both group. Table 2 illustrated comparisons between pyogenic and TB group.

**Table 2:** Comparisons between pyogenic and TB patients

Characters	Pyogenic (*N* = 9)	TB (*N* = 9)
Age	71.8	75.1
Sex (M:F)	1:8	1:8
Level		
T spine	3	7
L spine	9	5
1st surgery		
WBC(X1000)(/mL)	9.4	6.1
ESR(mm/h)	Inadequate data	Inadequate data
CRP(mg/L)	Inadequate data	Inadequate data
Revision surgery		
WBC (X1000)(/mL)	11.0	7.1
ESR (mm/h)	61.1	48.1
CRP (mg/L)	87.8	45.7
Revision method		
A	3	1
P	2	2
A + P	4	6
Neurologic Status (ASIA)		
E	7	4
D	1	2
C	1	2
B	0	1
A	0	0
Pre-revision VAS	7.2	7.7
Final VAS	3.1	2.6
Final activity		
Normal	5	2
Walker support	1	4
On wheelchair	1	2
Dead	2	1
CCI	1.9	1.4

Abbreviations: *TB* tuberculosis, *N* number, *M* male, *F* female, *T* thoracic, *L* lumbar, *WBC* white blood-cell count, *ESR* erythrocyte sedimentation rate, *CRP* C-reaction protein, *ASIA* American Spinal Cord Injury Association, *VAS* visual analog scale, *CCI* the Charlson Comorbidity index

## Discussion

The percutaneous transpedicular injection of cement into a vertebral body is a very minor procedure, but still carries a risk of developing postoperative infection. The reported incidence of infection after VP or KP is between 0 and 1% [12, 13]. In our study, with the largest study population of similar studies, the incidence was 0.32%, which was similar to that reported by Abdelrahman et al. (0.46%) [4].

Most bacteria or TB in this study were low-virulence organisms. We believe that these organisms already existed in the patients, and the infection process was triggered by the cement injection procedure, leading to the consequential results. Yu et al. described the first case of pyogenic spondylitis after VP, for a patient who had an episode of urinary tract infection just 1 week before VP [14]. Other infection sources, including cholecystitis, meningitis, or post-laminectomy wound infection, were thought to be associated with pyogenic spondylitis after VP [15, 16]. Under very rare conditions, even skin acne can cause post-VP spondylitis [17]. The first TB spondylitis after VP was reported in 2006; the authors suggested that the possible mechanism leading to the development of TB infection after VP was the release of mycobacteria from macrophages containing quiescent bacilli which migrated to the vertebrae of the VP. Ivo et al. and Kang et al. reported that cases with TB spondylitis after VP all had a pulmonary TB history [7, 9]. There were also five patients in the TB group of our study who had pulmonary TB when they were young. This suggests that TB bacilli could exist inside the body even after the completion of a course of medicine, and VP procedure might cause these TB bacilli to seed around the cemented vertebrae, leading to infection.

In addition to previous bacteraemia from UTI or other internal organs, common skin pathogens might also induce vertebrae infection after VP. In the current study, one patient had a *Staphylococcus aureus* infection and one developed Propionibacterium species infection; these two pathogens can be seen in normal skin. Propionibacterium species are common gram positive anaerobic flora on the skin or inside acne, and Propionibacterium acnes is the most virulent. Propionibacterium species infection usually involves total joint arthroplasty, particularly in hip or shoulder arthroplasty [18, 19]. Profound spine infection induced by Propionibacterium species is extremely rare; but latent infection by this particular specie is related to developing lumbar disc degeneration or herniation [20]. Before this study, there was only one case report of vertebral osteomyelitis following VP induced by Propionibacterium species, and the authors emphasised the importance of prophylactic antibiotics, even with this minimally invasive procedure [17].

For spontaneous infectious spondylitis, there are many distinguishing finding in radiologic, clinical and laboratory features between pyogenic and TB. A patient with pyogenic spondylitis generally demonstrates more previous invasive spinal procedures, preceding bacteraemia, more episodes of fever, higher elevation of CRP and ESR, a higher percentage of WBC counts over 10,000/mm3 and a fraction of neutrophils > 75%, and it is also more associated with chronic renal failure and liver cirrhosis. TB spondylitis is frequently associated with longer diagnostic delay, TB of other organs, more involvement of the thoracic spine, involvement of more spinal levels, and the presence of disc space sparing [21]. Similarly, in the present study, higher elevations of CRP, ESR and WBC counts were seen in the pyogenic group at the time of revision, however, there was more involvement of the thoracic spine, longer intervals between VP and revision surgery, and other organs affected by TB (lungs) were observed in the TB group.

Surgery is not always the initial treatment of choice for spontaneous infectious spondylitis. If the pathogenic organism is known, then conservative treatment with antibiotics or chemical agents for TB combined with motion restriction using a brace can be used to allowed recovery from infectious spondylitis. For those cases who have infectious spondylitis after VP, however, most of these patients will receive surgeries to relieve their intractable back pain, which is induced by unhealed fracture and infection; and some of these patients also has some degree of neurological impairment. The optimal surgical method for treating infection after VP was inconsistent in past literatures. Chen et al. demonstrated a case of pyogenic spondylitis after VP which could be successfully treated with percutaneous drainage followed by antibiotic-impregnated cement VP [22]. Laminectomy with pus drainage was successfully used to treat pyogenic or TB spine after VP, as reported by Soyuncu et al. [23]. Most spine surgeons reported in the literature advocated a combined anterior debridement/fusion and posterior fixation for this kind of patient [4, 5, 9, 14, 15, 24, 25]. In the current study, most patients (10 out of 18, 56%) also received combined anterior and posterior procedures for their infectious spondylitis. Because their infection site was not only at the level of VP but also had some degree spreading to an adjacent disc space or vertebrae, anterior wide debridement and fusion with structured bone graft was the main method to achieve adequate decompression and reconstruction, and following posterior instrumentation could provide immediately strong stability and enhance anterior fusion.

The incidence of infectious spondylitis after VP was low and most previous literatures only had cases reports or a small case series in their articles [4–9, 14, 15, 17, 24, 25]. Park et al. reported experiences of 11 cases after VP or kyphoplasty and reviewed the other 42 cases in 26 published English articles on PubMed research [26]. According to the study from Park et al., there were three patients in their 11 cases (27%) and 11 patients in the other 42 cases reviewed (26%) had no organism in culture tissue; *Staphylococcus aureus* (10 cases, 19%) and TB (10 cases, 19%) were the most pathogen in these 53 cases. In the present study, five patients were excluded because there was no definite causative organism obtained at their revision surgery. If these five cases were included into the present study, the negative tissue culture rate was 22% (5/23), which was similar to the results of Park et al.. However, the most pathogen in the present study was TB (9 cases), followed by Salmonella species (2 cases) and E coli (2 cases), the proportion of causative pathogen was different to that in Park’s study. Most cases of post-VP pyogenic spondylitis developed days to months after VP and most cases of post-VP TB spondylitis happened months to years after VP, this scenario was similar in our and Park’s study. In contrast to Park’s study, we had no cases of post-VP TB spondylitis happened within days to weeks after initial VP, but there were two cases among Park’s review; Park et al. thought the etiology of these two cases was pre-existed TB spondylitis and was misdiagnosed as simple osteoporosis compression fracture by theory of Chen et al [27].

## Conclusion

Although VP is a minimally invasive procedure, the possibility of postoperative infection should not be ignored. Infectious spondylitis after VP generally requires major salvage surgery to overcome problems of infection, nerve compression, and spinal instability. TB spondylitis after VP takes longer to detect with a lower degree of elevation in infection parameters, but both kinds of infection might lead to some residual disability, even in complete treatment. In addition to standard skin preparation and the administration of prophylactic antibiotics, surgeons should preoperatively consider immune status, UTI or other infection source within 6 months, and history of pulmonary TB to prevent infection after VP.

## Abbreviations

ASIA: American Spinal Injury Association impairment scale
CCI: Charlson Comorbidity Index
CRP: c reaction protein
ESR: erythrocyte sedimentation rate
TB: tuberculosis
UTI: urinary tract infection
VAS: Visual Analogue Scale
VP: vertebroplasty
WBC: white blood cell
